# Dementia risks identified by vocal features via telephone conversations: A novel machine learning prediction model

**DOI:** 10.1371/journal.pone.0253988

**Published:** 2021-07-14

**Authors:** Akihiro Shimoda, Yue Li, Hana Hayashi, Naoki Kondo

**Affiliations:** 1 Department of Public Health, McCann Healthcare Worldwide Japan Inc., Tokyo, Japan; 2 Department of Global Health Promotion, Tokyo Medical and Dental University, Tokyo, Japan; 3 Graduate School of Health Management, Keio University, Tokyo, Japan; 4 Department of Social Epidemiology and Global Health, Graduate School of Medicine and School of Public Health, Kyoto University, Kyoto, Japan; Vellore Institute of Technology: VIT University, INDIA

## Abstract

Due to difficulty in early diagnosis of Alzheimer’s disease (AD) related to cost and differentiated capability, it is necessary to identify low-cost, accessible, and reliable tools for identifying AD risk in the preclinical stage. We hypothesized that cognitive ability, as expressed in the vocal features in daily conversation, is associated with AD progression. Thus, we have developed a novel machine learning prediction model to identify AD risk by using the rich voice data collected from daily conversations, and evaluated its predictive performance in comparison with a classification method based on the Japanese version of the Telephone Interview for Cognitive Status (TICS-J). We used 1,465 audio data files from 99 Healthy controls (HC) and 151 audio data files recorded from 24 AD patients derived from a dementia prevention program conducted by Hachioji City, Tokyo, between March and May 2020. After extracting vocal features from each audio file, we developed machine-learning models based on extreme gradient boosting (XGBoost), random forest (RF), and logistic regression (LR), using each audio file as one observation. We evaluated the predictive performance of the developed models by describing the receiver operating characteristic (ROC) curve, calculating the areas under the curve (AUCs), sensitivity, and specificity. Further, we conducted classifications by considering each participant as one observation, computing the average of their audio files’ predictive value, and making comparisons with the predictive performance of the TICS-J based questionnaire. Of 1,616 audio files in total, 1,308 (81.0%) were randomly allocated to the training data and 308 (19.1%) to the validation data. For audio file-based prediction, the AUCs for XGboost, RF, and LR were 0.863 (95% confidence interval [CI]: 0.794–0.931), 0.882 (95% CI: 0.840–0.924), and 0.893 (95%CI: 0.832–0.954), respectively. For participant-based prediction, the AUC for XGboost, RF, LR, and TICS-J were 1.000 (95%CI: 1.000–1.000), 1.000 (95%CI: 1.000–1.000), 0.972 (95%CI: 0.918–1.000) and 0.917 (95%CI: 0.918–1.000), respectively. There was difference in predictive accuracy of XGBoost and TICS-J with almost approached significance (p = 0.065). Our novel prediction model using the vocal features of daily conversations demonstrated the potential to be useful for the AD risk assessment.

## Introduction

Identifying individuals at risk for Alzheimer’s disease (AD) in the prodromal phase might lead to early detection and alleviation of the burden of AD among patients and caregivers [1–5]. Due to difficulty in early diagnosis of AD related to cost and differentiate capability [6–8], it is necessary to identify low-cost, accessible, and reliable tools for identifying AD risk in the preclinical stage. Lately, an increasing amount of research has accumulated evidence about the greater accuracy and efficiency of prediction models using machine-learning algorithms such as random forest (RF) and extreme gradient boosting (XGBoost) as compared to the conventional schemes in medical classification problems [9, 10]. Indeed, recent studies have shown a number of successful applications of machine learning approaches to large-scale data for predicting disease, including AD, diabetes, metabolic syndrome, suicide, opioid overdose, or drug-resistant epilepsy, among others [11–16]. However, for AD risk prediction, the machine learning model developed in the previous study used large administrative health data (e.g., sociodemographic information, health profiles, and history of personal and family illness) and showed an Area Under the Curve (AUC) of 0.775, indicating much room for improvement.

The neurophysiology of AD provides a perspective for further improving AD risk prediction. AD patients represent the degree of deficits in specific cognitive constructs: neurophysiologic change following the progression of AD (e.g., presence of amyloid plaques, neurofibrillary tangles, and diffuse degeneration and atrophy of various parts of the cortex) can lead to changes in sensory perception and motor symptoms, resulting in impairment of spontaneous speech [17–19]. A stream of evidence has shown that AD patients are more likely to speak more slowly and with longer pauses, and spend more time finding the correct word, resulting in broken messages and lack of speech fluency [20–22]. These indicate the possibility of further developing further accurate prediction models using vocal features to identify AD risk [23, 24]. However, evidence about AD prediction using vocal features remains scarce.

The purpose of the present study is to 1) develop a novel machine learning prediction model to identify AD risk using only vocal features collected from daily conversations via telephone, and 2) evaluate the predictive performance of the model by comparing results of multiple machine learning algorithms with conventional cognitive tests. We believe that if the developed model using daily conversation voice data can accurately predict AD risk, it will have a significant impact on early detection and diagnosis among the general older adult population in that we can guide those who are in the earliest stages of AD to engage in care-seeking behavior.

## Materials and methods

### Study design

The present study is a retrospective analysis of voice data and conventional cognitive test data among individuals ages 65 and older who participated in a program aimed at dementia prevention in a Japanese local city. Using these data, we developed prediction models and compared predictive accuracy with that of a conventional cognitive test. The present study was approved by the Institutional Review Board of Kyoto University (examination number: R2721). This paper adhered to the Transparent Reporting of a Multivariable Prediction Model for Individual Prognosis or Diagnosis statement (TRIPOD), which was proposed for the reporting of predictive models [25].

### Data source and study population

**Fig 1:**
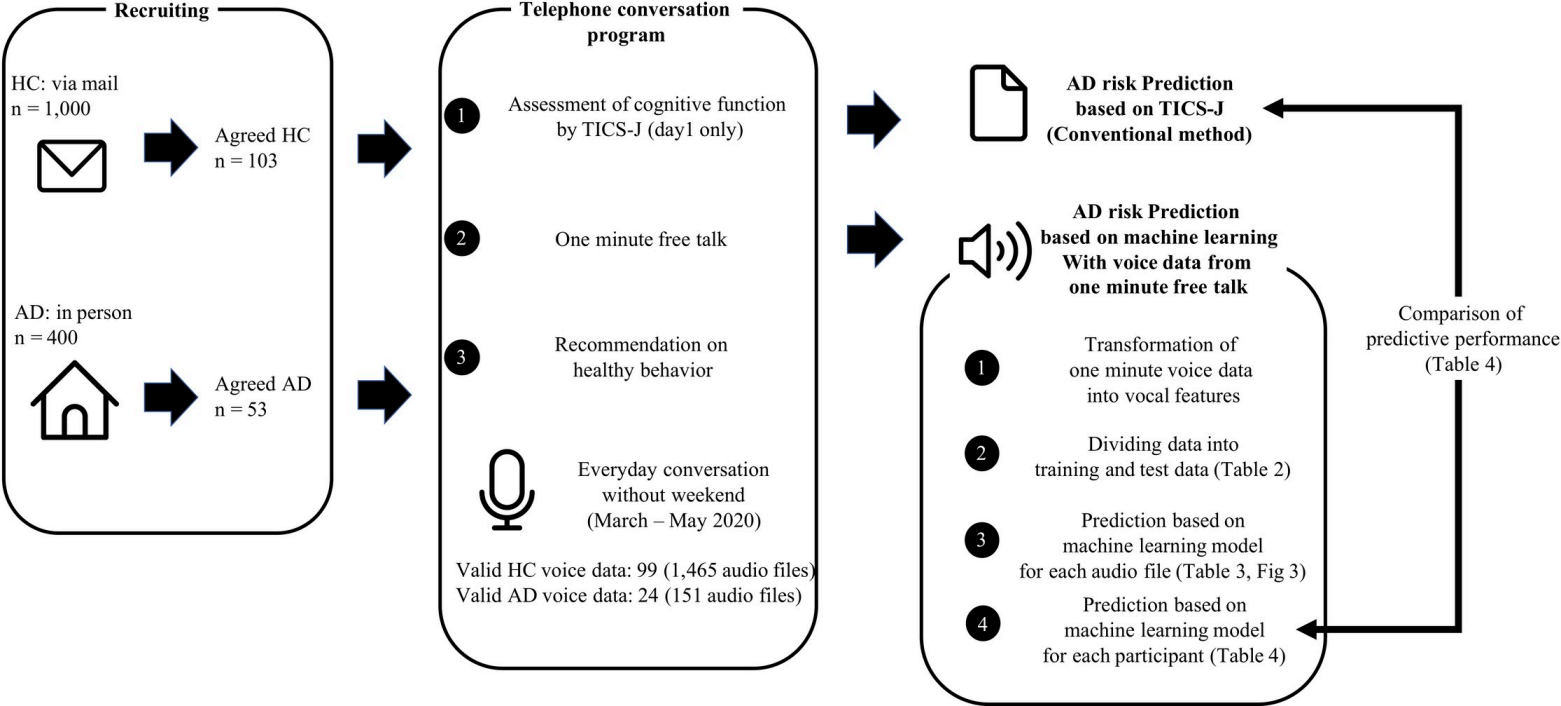
The whole process in the present study.

The data in the present study was gathered from Hachioji City, Tokyo in the spring of May 2020 (Longitude: 35.6663; Latitude: 139.3158). The total population was 576,608 as of August 1, 2020. The study population included residents in the city aged 65 or older who agreed to participate in a telephone conversation program, with an Artificial Intelligence (AI) computer program, aimed at improving healthy diet, physical activity, and social participation for prevention of dementia. The city recruited both HC and AD patients: For HC, participants were those who, after receiving an invitation letter about the program from Hachioji City via mail (sent to 1,000 people randomly), voluntarily agreed to participate. Of the 1,000 people contacted, 103 agreed to participate (10.3%). For AD patients, participants were those who volunteered or whose families agreed to participate after an in-person invitation to the program from Hachioji City, and who currently used a day service center (a welfare facility designed to care for senior citizens with AD). Among 400 patients, 53 agreed to participate (13.2%). Thus, the final number of participants for HC and AD patients were 103 and 53, respectively (Fig 1).

### The contents of the dementia prevention program

The dementia prevention program conducted by the city consisted of a telephone conversation with AI that covered the following contents: 1) assessment of cognitive function based on the Japanese version of the Telephone Interview for Cognitive Status (TICS-J) [26] (day 1 only); 2) asking participants to talk about daily life for one minute using questions such as “What did you do yesterday?”; 3) recommendations on healthy behavior including healthy diet, physical activity, and social participation based on topic recognition by AI analysis of participants’ voice patterns. The AI built into the program for voice recognition was developed by Softfront Japan (Tokyo, Japan) and McCann Health Japan (Tokyo, Japan). The program included 1–2 months of weekday telephone conversations. This telephone conversation program was adopted by Hachioji City because a service via telephone is highly accessible for every resident and does not require preparation of any additional devices. All that participants needed in advance was a registered telephone number and their name.

### Study protocol

We used data obtained from the telephone conversation program conducted between March 2020 to May 2020. The data we received came from HC and AD patients that had at least one valid audio file, numbering 99 and 24, respectively. For all patients we extracted 1) the results of the assessment of cognitive function with a questionnaire based on TICS-J, 2) voice data of participants, especially the 1-minute talk portion, and 3) a binary variable indicating whether they were an HC (0) or AD (1) patient. Whereas we got one result of TICS-J based questionnaire for each participant, we obtained multiple recordings of the 1-minute talk for each participant because the telephone conversation program consisted of 1–2 months of daily weekday telephone conversations, resulting in multiple recording files for each participant, with an average of 13.1 (Standard deviation: 7.6). The data-processing steps, as well as other processes in the present study, are shown in Fig 1.

### Dataset creation and definitions

#### AD data (outcome)

Our study consisted of 99 HC and 24 AD patients. As stated above, HC and AD patients were recruited in different ways. Whereas HC patients were recruited via mail, AD patients were recruited in person given the difficulty of explaining the program. AD patients were previously diagnosed using National Institute on Aging-Alzheimer’s Association (NIA-AA) criteria [27] and/or the Diagnostic and Statistical Manual of Mental Disorders, 5^th^ ed. (DSM-5) [28] before the program. We had to exclude patients with severe AD from recruitment as they could not participate in the telephone conversation program due to limitations in cognitive capacity. Thus, those included in the telephone conversation program may represent patients with mild/moderate AD or mild cognitive impairment (MCI). We coded 1 if a participant was an AD patient, and otherwise coded a 0, and used this binary variable as an outcome for prediction.

#### Vocal feature extraction

The voice data used for model prediction were collected through the telephone conversation program: each participant was asked to have a nested conversation with an AI computer program. The conversation consisted of a greeting, a task that asked the participant to describe what he or she did yesterday with as much detail as they could in one minute, and closed with recommendation of health behavior and scheduling of the next call. The participant’s response to the task was the only part of the conversation recorded and used for future analysis. The reason we used this one-minute task is that in many validated questionnaires for screening of dementia like MMSE, memory and the ability to express one’s thoughts are crucial elements that have high discriminating ability in screening for dementia [29].

After recording, vocal features were extracted using the open software tool PRAAT [30]. PRAAT has been widely used for phonetic analysis worldwide, and it enables us to extract various vocal features from recorded speech. In our study, for each voice recorded we extracted all possible information including 1) the start and end time of all sounding and silent intervals, 2) intensity by every 0.01 of a second, 3) pitch by every 0.02 of a second, and 4) center of gravity, skewness, kurtosis, and standard deviation. All four features were written into four separate txt files by running a PRAAT scripting language. Then, python scripts were developed to read all txt files and generate variables used for model prediction. Based on previous studies, we made some modifications and ultimately generated 60 vocal variables [24, 31]. In this process, intensity and pitch were further used to generate the “derivatives”, i.e. the change in intensity or pitch every time interval, by subtracting the intensity or pitch at the previous time point from the present time point. For intensity and pitch, as well as their “derivatives”, we generated the following variables: mean, median, minimum, maximum, 0.15 percentile, 0.85 percentile, standard deviation, skewness, and kurtosis. For example, the median value for the “derivatives” of pitch means the median value of the person’s changes in pitch across (altogether 4*9 = 36 variables). For sounding and silent intervals, in addition to the above variables, we added the sum of length of both types of intervals (altogether 2*10 = 20 variables). For spectrum, we computed center of gravity, skewness, kurtosis, and standard deviation as another four variables. All the vocal features created by this process are shown later (Tables 2 and S2). We finally obtained 1,465 and 151 audio files for HC and AD patients with averages of 15.8±5.9 and 5.0±6.2 files for each participant, respectively.

### Model generation

**Fig 2:**
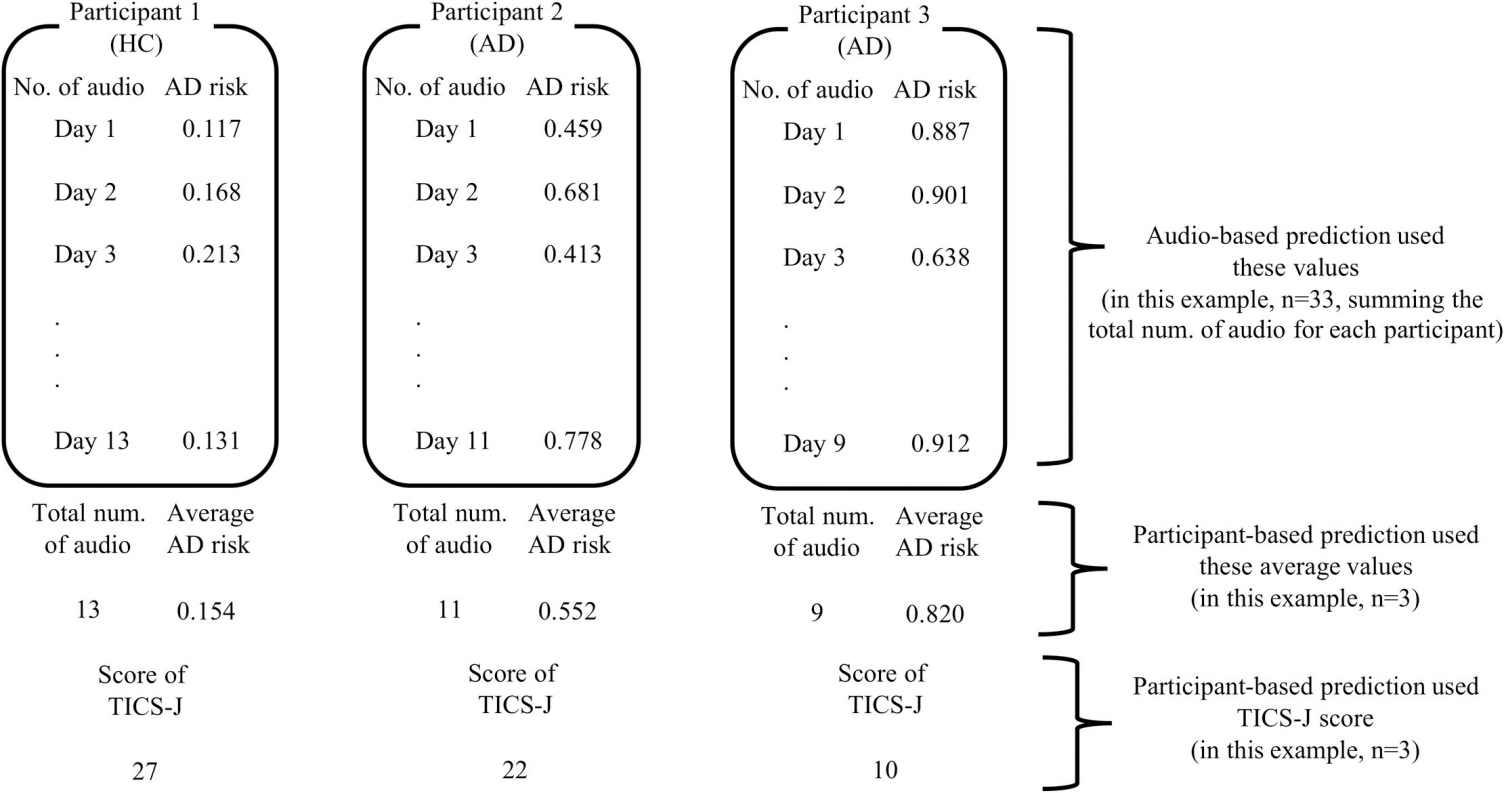
An illustration of the difference between audio-based prediction and participant-based prediction.

We developed three machine-learning prediction models, applying the extreme gradient boosting (XGBoost) [32], random forest (RF) [33], and logistic regression (LR) [34]. We computed these models using the “caret” package in R (Version 4.0.2) [35]. These algorithms may be basic but are well-accepted to deal with predictive tasks regardless of the field. All models were trained and tested on a randomly partitioned 80/20 percentage split of the dataset. We conducted cluster randomized partition so that audio files of the same participant were not included in both training data and validation data. The models for ‘audio-based prediction’ were developed using each audio file as one observation (HC: n = 1,465, AD: n = 151). For ‘participant-based prediction’, we averaged each audio’s predictive value for every participant (HC: 99, AD: 24). Further, we also developed a TICS-J based questionnaire model, with a validated classification method using cognitive test, for ‘participant-based prediction’. We described an illustration of difference between ‘audio-based prediction’ and ‘participant-based prediction’ in Fig 2 (Note that the number in Fig 2 is just an illustration and not the real number).

#### Extreme gradient boosting model

In short, XGBoost is an ensemble of classification and regression trees (CART) [36]. A classification/regression tree is trained based on an ensemble of previously trained classification/regression trees in order to improve predictive accuracy through the minimization loss function: in other words, the algorithm’s computation of boosting is built on a number of weak classifiers. As each CART assigns a real score to each leaf (outcome), the predictive scores for a CART are summed up to calculate the final score and assessed through additive functions. XGBoost has been widely accepted as the one of the models with the most impressive predictive accuracy [37, 38].

#### Random forest model

RF is an ensemble-based method that uses multiple decision trees like XGBoost, but it is different in that RF computes predictive scores by averaging the vote for each tree, iterating over all trees in the ensemble [33]. Each tree is developed from a random subset of the dataset through a bagging method. As each tree tends to overfit in a different way, random decision forests can correct for this overfitting by voting. RF is frequently used in research and business settings as it requires few configurations and generates reasonable predictions for a wide range of data.

#### Logistic regression model

LR is a commonly used statistical method for a variety of classification tasks [34]. LR employs a logistic function to model a binary outcome represented by ‘0’ or ‘1’. The model assumes the log-odds for the outcome coded ‘1’ is a linear combination of independent variables. Thus, LR is an extension of the linear regression model for classification. LR has advantages in that it is easier to compute, interpret, and efficient to train, whereas disadvantages include the assumption of linearity between outcome and independent variables.

#### TICS-J based questionnaire model

In addition to machine learning models, we developed the scoring model using the TICS-J based questionnaire, assessing the cognitive function of participants through telephone interviews [26]. TICS-J is the Japanese version of TICS, which consists of an 11-item screening test that was developed for assessing cognitive function in AD patients who are unwilling or unable to be examined in person [39]. TICS has been widely accepted for measuring cognitive function and performance, and was significantly correlated with a Mini-Mental State Examination (MMSE) score (r = 0.86, p<0.001) [39]. TICS-J also showed high performance in differentiating AD patients from HC with a sensitivity of 98.0% and specificity of 90.7%, and also significantly correlated with MMSE score (r = 0.86, p<0.001) [26]. We adopted the cognitive function test via telephone interview based on TICS-J (S1 Table). As the original version of TICS-J is supposed to be conducted with a human operator, the setting of the cognitive test was different with our program: the telephone conversation program was conducted between the AI computer program and participants, leading to some changes in the questionnaire, taking into account the limitations on voice recognition and communication of AI. For instance, question 6: “One hundred minus 7 equals what?” should be stopped at 5 serial subtractions in the original version, whereas we stopped at 2 serial subtractions. Also, question 7: “What do people usually use to cut paper?” and “How many things are in a dozen?” should be followed by the subsequent two questions “What do you call the prickly green plant that lives in the desert?” and “What is tofu made from?” in the original version, yielding 4 points in total. We needed to cut parts of questions 6 and 7 in order to save time for the entire interview and avoid impairing the whole questionnaire, because we found that many participants could not last through the long interviews with AI and hung up before it was completed. The total score for the questionnaire was calculated by a human data administrator, using the recording for each participant. The score ranged from 0 to 36 and used the cognitive ability measure as a continuous variable. Those scored below 25 were classified as AD, according to the threshold of TICS-J.

### Tuning of parameters

We needed to consider the fine-tuning of several parameters when adopting XGBoost, RF, and LR. The parameters for our prediction models were set through a grid search, a method for optimization of parameters using combinations of each parameter. We trained 10 different models with 90% of the training data and tested them with the remaining 10% for each grid search process. The results of the grid search for the prediction models are shown in Table 1. In the end, we developed the models for prediction using these parameters.

**Table 1:** Parameter values in each model.

Model	Parameter	Value
XGBoost	nrounds	150
	max_depth	1
	eta	0.3
	gamma	0
	colsample_bytree	0.8
	min_child_weight	1
	subsample	0.6666667
Random forest	mtry	192
Logistic regression	alpha	0.1
	lambda	0.004858939

nrounds = Number of iterations; max_depth = The maximum depth of variable interactions; eta = Control of learning rate; gamma = Minimum loss reduction required to make a further partition on a leaf node of the tree; colsample_bytree = Subsample ratio of columns when constructing each tree; min_child_weight = Minimum sum of instance weight (hessian) needed in a child; num.trees = Number of trees; mtry = Number of variables to possibly split at in each node; alpha = Learning rate and controls how much the coefficients (and therefore the model) changes or learns each time it is updated; lambda = regularization rate aimed at balancing between simplicity and training-data fit.

### Model comparison

We carried out two types of model comparison: one based on ‘audio-based prediction’, and the other on ‘participant-based prediction’.

#### Audio-based prediction

First, we made a comparison between models with machine-learning (XGBoost, RF, and LR) using each audio file as one observation. We evaluated the predictive performance of the developed models by describing the receiver operating characteristic (ROC) curve, calculating the areas under the curve (AUCs), sensitivity, and specificity. Subsequently, we compared the predictive performance of developed models using the chi-squared test proposed by DeLong [40]. We determined the threshold for each model using the Youden index that maximizes Sensitivity + Specificity– 1 [41].

#### Participant-based prediction

Subsequently, we made a comparison between models with machine-learning (XGBoost, RF, and LR) and the TICS-J based questionnaire using each participant as one observation. As stated above, we yielded 1,465 and 151 audio files for HC and AD patients with averages of 15.8±5.9 and 5.0±6.2 files for each participant, respectively. By regarding each audio file as one observation, our development of the prediction models made a tacit assumption that each audio file is independent in terms of vocal characteristics, which is not actually the case. Although we made sure that the audio file of the same participant would not be included in both the training data and validation data, it raised a potential problem. Thus, as a further evaluation of our prediction models, we conducted additional analysis to measure the predictive accuracy for each participant, not for each audio file. Although limited sample size meant that we could not develop a model based on each participant, we instead conducted a participant-based prediction by computing the average of the predictive value among their multiple audio files, and used this for the validation data. We already described this concept in Fig 2 as an illustration. The metrics used for comparison were the same as the audio-based prediction. All of the analyses were conducted in R (Version 4.0.2) [42].

## Results

### Descriptive characteristics of participants

Our final participants consisted of 99 HC and 24 AD patients, yielding 1,465 and 151 audio files for each group. Of 1,616 audio files in total, 1,308 (81.0%) were randomly allocated to the training data and 308 (19.1%) to the validation data. Among those, 123 (9.4%) of the training data and 28 (9.1%) of the validation data were audio files of AD patients (S2 Table). On a participant basis, 99 (80.5%) were allocated to the training data and 24 (19.5%) to the validation data. The mean age±SD for training data and validation data were 74.6±6.6 and 76.7±7.5, respectively. The proportion of females was 57.0% in the training data and 54.1% in the validation data. The AD patients in the training data and validation data were 24 (24.2%) and 6 (25.0%), respectively (Table 2).

**Table 2:** Descriptive statistics of demographic and vocal data for participants of each group.

Variable	Training data, mean (SD) n = 99	Validation data, mean (SD) n = 24	P-value
Age	74.6 (6.6)	76.7 (7.5)	0.223
Gender (female) n,%	53 (57.0%)	13 (54.1%)	0.428
Vocal features			
Silent: the duration when the participant is not speaking (second)			
silent_sum	26.4 (12.6)	25.1 (7.3)	0.514
silent_mean	0.8 (0.5)	0.8 (0.3)	0.640
silent_median	0.3 (0.1)	0.3 (0.2)	0.520
silent_minimum	0.1 (0)	0.1 (0)	0.414
silent_maximum	5.9 (4.5)	5.2 (2.1)	0.215
silent_15percentile	0.1 (0)	0.1 (0)	0.482
silent_85percentile	1.4 (0.7)	1.5 (0.6)	0.529
silent_standard deviation	1.3 (1.1)	1.2 (0.5)	0.255
silent_skewness	2.5 (0.8)	2.4 (0.6)	0.589
silent_kurtosis	7.6 (5.2)	6.9 (3.5)	0.468
Sounding: the duration when the participant is speaking (second)			
sounding_sum	19.1 (10.8)	17.7 (6.9)	0.436
sounding_mean	0.6 (0.1)	0.5 (0.1)	0.541
sounding_median	0.4 (0.1)	0.4 (0.1)	0.675
sounding_minimum	0.1 (0)	0.1 (0)	0.865
sounding_maximum	1.9 (0.5)	1.8 (0.5)	0.767
sounding_15percentile	0.2 (0)	0.2 (0.1)	0.722
sounding_85percentile	1 (0.2)	0.9 (0.3)	0.296
sounding_standard deviation	0.5 (0.1)	0.4 (0.1)	0.504
sounding_skewness	1.2 (0.3)	1.3 (0.3)	0.365
sounding_kurtosis	1.4 (1.1)	1.7 (1.4)	0.388
Pitch: Voice pitch of the participant (Hz)			
pitch_mean	190.2 (35.4)	194.8 (29.1)	0.510
pitch_median	176.6 (40.7)	179.9 (37)	0.705
pitch_minimum	70.8 (22.9)	72.9 (19.1)	0.643
pitch_maximum	522.8 (68.4)	525 (68)	0.886
pitch_15percentile	144.2 (34.6)	146.5 (30.5)	0.751
pitch_85percentile	234.9 (48.8)	241.8 (34.8)	0.429
pitch_standard deviation	63.6 (20.5)	65.4 (19.2)	0.688
pitch_skewness	2.4 (1.4)	2.2 (1.1)	0.425
pitch_kurtosis	12.2 (10.4)	10 (7.1)	0.221
Pitch difference: Amount of change in voice pitch every 0.01 seconds (Hz)			
pitch_d_mean	-0.4 (0.7)	-0.3 (0.7)	0.314
pitch_d_median	-1 (0.4)	-1 (0.4)	0.704
pitch_d_minimum	-146.3 (64.8)	-153.9 (59.6)	0.583
pitch_d_maximum	204.4 (78.9)	215.4 (85.2)	0.570
pitch_d_15percentile	-5.3 (1.4)	-5.2 (1.3)	0.698
pitch_d_85percentile	3.4 (1.4)	3.5 (1.3)	0.659
pitch_d_standard error	16.1 (5.5)	17.5 (5.6)	0.283
pitch_d_skewness	3.5 (3.6)	3.4 (3.2)	0.822
pitch_d_kurtosis	118.5 (58.5)	111.9 (40.4)	0.518
Intensity: Voice intensity of the participant (dB)			
intensity_mean	74.8 (4.2)	75.1 (5.3)	0.772
intensity_median	76.4 (4.6)	76.7 (5.7)	0.796
intensity_minimm	34.6 (7.4)	35.9 (5.3)	0.332
intensity_maximum	89.2 (3)	89.5 (3.8)	0.717
intensity_15percentile	66.8 (3.6)	67.1 (4.9)	0.786
intensity_85percentile	82.8 (4.6)	83.3 (5.6)	0.686
intensity_standard deviation	8.7 (0.9)	8.8 (0.9)	0.438
intensity_skewness	-1.2 (0.4)	-1.2 (0.3)	0.569
intensity_kurtosis	2.8 (3.6)	2.2 (0.9)	0.145
Intensity difference: Amount of change in voice intensity every 0.01 seconds (dB)			
intensity_d_mean	0 (0)	0 (0)	0.481
intensity_d_median	-0.1 (0)	-0.1 (0)	0.577
intensity_d_minimum	-11.3 (3.3)	-11 (1.4)	0.417
intensity_d_maximum	15 (3.8)	14.2 (1.6)	0.109
intensity_d_15percentile	-1.8 (0.3)	-1.8 (0.4)	0.807
intensity_d_85percentile	1.6 (0.3)	1.7 (0.5)	0.447
intensity_d_standard deviation	2.6 (0.3)	2.6 (0.4)	0.785
intensity_d_skewness	0.7 (0.3)	0.6 (0.3)	0.264
intensity_d_kurtosis	5.7 (8.3)	4.4 (1.4)	0.135
Spectrum (Hz)			
spectrum_center of gravity	599.8 (136.1)	621.6 (145)	0.507
spectrum_standard deviation	461.4 (125.4)	480.9 (116.3)	0.474
spectrum_skewness	3.1 (0.9)	3 (0.8)	0.296
spectrum_kurtosis	14.8 (9.4)	12.3 (7)	0.154
AD patients n,%	24 (24.2%)	6 (25.0%)	0.940

#### The comparison results of audio-based prediction

**Fig 3:**
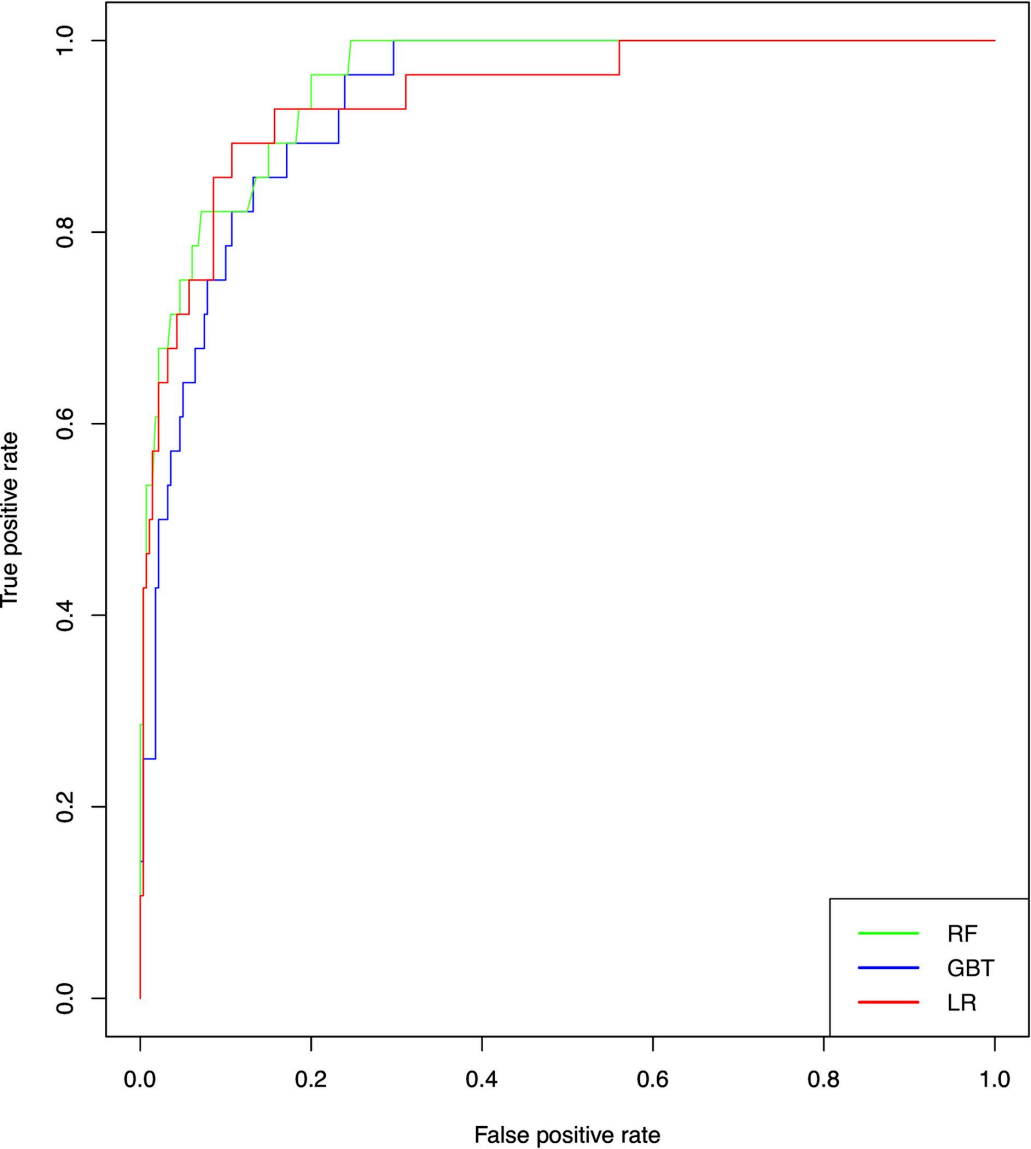
Receiver operating characteristic (ROC) curves in the models for predicting AD. RF = Random Forest; XGBoost = Extreme Gradient Boosting; LR = Logistic Regression.

The predictive performance of developed machine-learning models built for each audio file are represented in Table 3, and the ROC curves for each model are shown in Fig 3. The AUC for XGboost, RF, and LR were 0.863 (95% confidence interval [CI]: 0.794–0.931), 0.882 (95% CI: 0.840–0.924), and 0.893 (95%CI: 0.832–0.954), respectively. The LR model achieved the best AUC, but there were no significant differences between the performances of the models.

**Table 3:** Predictive performance of the models built for each audio file for predicting AD.

Model	AUC	95%CI	Sensitivity	Specificity	Threshold (range)	Prob > χ^2^^†^
XGBoost	0.863	0.794–0.931	0.857	0.867	0.04271042 (0–1)	
RF	0.882	0.840–0.924	0.964	0.800	0.1316 (0–1)	0.5409
LR	0.893	0.832–0.954	0.893	0.893	0.09859087 (0–1)	0.5367

XGBoost = Extreme Gradient Boosting; RF = Random Forest; LR = Logistic Regression; AUC = Area Under the Curve; 95%CI = 95% Confidence Interval.

† Compared with XGBoost model.

#### The comparison results of participant-based prediction

Subsequently, the predictive performance of developed machine-learning models and cognitive test (TICS-J based questionnaire) built for each participant are represented in Table 4. The AUC for XGboost, RF, LR, and cognitive test were 1.000 (95%CI: 1.000–1.000), 1.000 (95%CI: 1.000–1.000), 0.972 (95%CI: 0.918–1.000) and 0.917 (95%CI: 0.918–1.000), respectively. There were no significant differences between the models, although the comparison of XGBoost and the cognitive test showed p = 0.065, indicating almost approached significance.

**Table 4:** Predictive performance of the models built for each participant for predicting AD.

Model	AUC	95%CI	Sensitivity	Specificity	Threshold (range)	Prob > χ2†
XGBoost	1.000	1.000–1.000	1.000	1.000	0.189 (0–1)	
RF	1.000	1.000–1.000	1.000	1.000	0.211 (0–1)	1.000
LR	0.972	0.918–1.000	1.000	0.944	0.144 (0–1)	0.317
TICS-J based questionnaire	0.917	0.828–1.000	1.000	0.833	20 (0–36)	0.065

XGBoost = Extreme Gradient Boosting; RF = Random Forest; LR = Logistic Regression; AUC = Area Under the Curve; 95%CI = 95% Confidence Interval.

† Compared with XGBoost model.

## Discussion

The machine learning models we developed, which were based on models built for each audio file, did well at classifying the audio files of AD and HC patients. Further, when the average of the predicted values of each audio file was summarized for each participant, the XGBoost model demonstrated performance comparable to cognitive tests, with almost approached significance.

Our finding is in line with previous studies. There is growing consensus that the presence of language deficits could be a part of clinical manifestation of AD and MCI, and suggestion that assessment of language production might be able to represent a unique opportunity for early detection of AD [43]. There have been several preceding works representing the performance of prediction models to differentiate AD from HC using acoustic and language features [44, 45]. Our results further supported this line of evidence. Moreover, our novel prediction model is significant in the sense that it showed strong performance even though it was developed solely from vocal features: previous studies tended to use other features such as demographic information in addition to vocal features to achieve high predictive accuracy [45]. Another strength of our study is that our vocal features consisted of daily conversations, not NPT in a clinical setting. Our achievement in predicting AD well using only vocal features from daily conversation indicates the possibility of developing a pre-screening tool for AD among the general population that is more accessible and lower-cost.

Our prediction models averaging the predictive value of each audio file for each participant showed even stronger performance than those built for each audio file. Although we need to interpret this result with caution, it might have potential for further robust prediction of AD by obtaining multiple audio files of daily conversations for each participant. Nevertheless, we are currently not sure if this method of compressing predictive values by arithmetic mean is appropriate for predicting AD risk in datasets other than those we already obtained. Although this idea, averaging the multiple predictive value of each weak learner, is widely accepted as a part of machine-learning algorithm such as random forest [33], further study is required to validate our models and whether or not they predict AD risk well for completely new subjects.

The findings of our study can create the opportunity for building new tools to identify AD risk by using only vocal features obtained from daily conversations via telephone, as a pre-screening method among the general population. It might enable and drive early detection and diagnosis of dementia, including AD, in the sense that the tool can be used not only by healthcare professionals in a clinical setting, but also the general population at home. As internet and mobile technology further improves, our prediction model can also be easily installed on a variety of user interfaces, such as websites, mobile apps, or the Internet of Things (IoT). Indeed, there have been several recent research assessments of cognitive health showing remarkable accuracy, based on machine learning algorithms using data from smart homes or smartphones [46, 47]. Given that many individuals who meet the criteria for dementia are estimated to be undiagnosed [4], providing the opportunity to assess their AD risk would lead to further care-seeking behavior and subsequent early detection among those “unconscious” people.

### Limitations

There are several limitations to our study. First, the outcome variable we used was binary (AD or HC), ignoring various features among AD patients. For example, speech characteristics may differ between advanced AD patients and MCI patients. Future research is expected to build prediction models for both advanced AD and MCI based on more detailed diagnostic information. Second, our small sample size to some extent limited our predictive power. Third, the quality of audio differed depending on the participants and time, raising the possibility that this affected the performance of the prediction models. Fourth, the questionnaire based on TICS-J that was used to assess cognitive function was conducted between the AI computer program and participants; the limited speech recognition ability of the AI computer program can affect the validity of obtained results. Fifth, we only relied on superficial vocal features such as pitch, intensity, etc. in the analysis, raising the possibility of loss of information and insufficient audio feature extraction. Further research could include natural language processing of speech content and sentence structure analysis in order to reduce information loss and increase model prediction performance. In practice, this device could be helpful to use as a gatekeeper of early diagnosis of AD through potential patients’ daily life. For the final diagnosis, it is necessary to also consider other symptoms, along with medical doctors’ judgements.

## Conclusions

Prediction models based on machine learning algorithms that use only vocal features from daily conversations showed strong predictive performance of AD risk, and were compatible with existing cognitive tests. This opens the possibility of developing new accessible, low-cost pre-screening tools for AD risk among the general population, outside a clinical setting.

## Supporting information

S1 Table The questionnaire for assessing cognitive function based on TICS-J. (DOCX)

S2 Table Descriptive statistics of demographic and vocal data for each group on audio file basis. (DOCX)

## References

[1] Harada K, Lee S, Shimada H, Lee S, Bae S, Anan Y, et al. Psychological predictors of participation in screening for cognitive impairment among community-dwelling older adults. Geriatr Gerontol Int. 2017;17: 1197–1204. doi: 10.1111/ggi.12841 27427234

[2] Bradford A, Upchurch C, Bass D, Judge K, Snow AL, Wilson N, et al. Knowledge of documented dementia diagnosis and treatment in veterans and their caregivers. Am J Alzheimers Dis Other Demen. 2011;26: 127–133. doi: 10.1177/1533317510394648 21273206 PMC10845555

[3] Duboisa B, Padovanib A, Scheltensc P, Rossid A, Agnello GD. Timely diagnosis for alzheimer’s disease: A literature review on benefits and challenges. J Alzheimer’s Dis. 2015;49: 617–631. doi: 10.3233/JAD-150692 26484931 PMC4927869

[4] Bunn F, Goodman C, Sworn K, Rait G, Brayne C, Robinson L, et al. Psychosocial Factors That Shape Patient and Carer Experiences of Dementia Diagnosis and Treatment: A Systematic Review of Qualitative Studies. PLoS Med. 2012;9. doi: 10.1371/journal.pmed.1001331 23118618 PMC3484131

[5] Boise L, Camicioli R, Morgan DL, Rose JH, Congleton L. Diagnosing dementia: Perspectives of primary care physicians. Gerontologist. 1999;39: 457–464. doi: 10.1093/geront/39.4.457 10495584

[6] Bradford A, Kunik ME, Schulz P, Williams SP, Singh H. Missed and delayed diagnosis of dementia in primary care: Prevalence and contributing factors. Alzheimer Disease and Associated Disorders. NIH Public Access; 2009. pp. 306–314. doi: 10.1097/WAD.0b013e3181a6bebc 19568149 PMC2787842

[7] Eichler T, Thyrian JR, Hertel J, Michalowsky B, Wucherer D, Dreier A, et al. Rates of formal diagnosis of dementia in primary care: The effect ofscreening. Alzheimer’s Dement Diagnosis, Assess Dis Monit. 2015;1: 87–93. doi: 10.1016/j.dadm.2014.11.007 27239495 PMC4876881

[8] Parmar J, Dobbs B, McKay R, Kirwan C, Cooper T, Marin A, et al. Diagnosis and management of dementia in primary care: Exploratory study. Can Fam Physician. 2014;60: 457–465. Available: /pmc/articles/PMC4020652/?report = abstract 24829010 PMC4020652

[9] Süt N, Şenocak M. Assessment of the performances of multilayer perceptron neural networks in comparison with recurrent neural networks and two statistical methods for diagnosing coronary artery disease. Expert Syst. 2007;24: 131–142. doi: 10.1111/j.1468-0394.2007.00425.x

[10] Kurt I, Ture M, Kurum AT. Comparing performances of logistic regression, classification and regression tree, and neural networks for predicting coronary artery disease. Expert Syst Appl. 2008;34: 366–374. doi: 10.1016/j.eswa.2006.09.004

[11] Park JH, Cho HE, Kim JH, Wall MM, Stern Y, Lim H, et al. Machine learning prediction of incidence of Alzheimer’s disease using large-scale administrative health data. npj Digit Med. 2020;3: 1–7. doi: 10.1038/s41746-019-0211-0 32258428 PMC7099065

[12] Farran B, Channanath AM, Behbehani K, Thanaraj TA. Predictive models to assess risk of type 2 diabetes, hypertension and comorbidity: Machine-learning algorithms and validation using national health data from Kuwait-a cohort study. BMJ Open. 2013;3: e002457. doi: 10.1136/bmjopen-2012-002457 23676796 PMC3657675

[13] Shimoda A, Ichikawa D, Oyama H. Prediction models to identify individuals at risk of metabolic syndrome who are unlikely to participate in a health intervention program. Int J Med Inform. 2017. doi: 10.1016/j.ijmedinf.2017.12.009 29425640

[14] Choi SB, Lee W, Yoon JH, Won JU, Kim DW. Ten-year prediction of suicide death using Cox regression and machine learning in a nationwide retrospective cohort study in South Korea. J Affect Disord. 2018;231: 8–14. doi: 10.1016/j.jad.2018.01.019 29408160

[15] Lo-Ciganic WH, Huang JL, Zhang HH, Weiss JC, Wu Y, Kwoh CK, et al. Evaluation of Machine-Learning Algorithms for Predicting Opioid Overdose Risk Among Medicare Beneficiaries With Opioid Prescriptions. JAMA Netw open. 2019;2: e190968. doi: 10.1001/jamanetworkopen.2019.0968 30901048 PMC6583312

[16] An S, Malhotra K, Dilley C, Han-Burgess E, Valdez JN, Robertson J, et al. Predicting drug-resistant epilepsy—A machine learning approach based on administrative claims data. Epilepsy Behav. 2018;89: 118–125. doi: 10.1016/j.yebeh.2018.10.013 30412924 PMC6461470

[17] Ranasinghe KG, Gill JS, Kothare H, Beagle AJ, Mizuiri D, Honma SM, et al. Abnormal vocal behavior predicts executive and memory deficits in Alzheimer’s disease. Neurobiol Aging. 2017;52: 71–80. doi: 10.1016/j.neurobiolaging.2016.12.020 28131013 PMC5359035

[18] Ballard C, Gauthier S, Corbett A, Brayne C, Aarsland D, Jones E. Alzheimer’s disease. The Lancet. Elsevier B.V.; 2011. pp. 1019–1031. doi: 10.1016/S0140-6736(10)61349-9 21371747

[19] Bayles K. MCI and Alzheimer’s Dementia: Clinical Essentials for Assessment and Treatment of Cognitive-Communication Disorders. 1st edition. Plural Publishing, Inc.; 2013. Available: https://www.amazon.com/MCI-Alzheimers-Dementia-Essentials-Cognitive-Communication/dp/1597565180

[20] Martínez-Sánchez F, Meilán JJG, García-Sevilla J, Carro J, Arana JM. Oral reading fluency analysis in patients with Alzheimer disease and asymptomatic control subjects. Neurol (English Ed. 2013;28: 325–331. doi: 10.1016/j.nrl.2012.07.012 23046975

[21] Hoffmann I, Nemeth D, Dye CD, Pákáski M, Irinyi T, Kálmán J. Temporal parameters of spontaneous speech in Alzheimer’s disease. Int J Speech Lang Pathol. 2010;12: 29–34. doi: 10.3109/17549500903137256 20380247

[22] Roark B, Mitchell M, Hosom JP, Hollingshead K, Kaye J. Spoken language derived measures for detecting mild cognitive impairment. IEEE Trans Audio, Speech Lang Process. 2011;19: 2081–2090. doi: 10.1109/TASL.2011.2112351 22199464 PMC3244269

[23] Boschi V, Catricalà E, Consonni M, Chesi C, Moro A, Cappa SF. Connected speech in neurodegenerative language disorders: A review. Frontiers in Psychology. Frontiers Research Foundation; 2017. p. 269. doi: 10.3389/fpsyg.2017.00269 28321196 PMC5337522

[24] König A, Satt A, Sorin A, Hoory R, Toledo-Ronen O, Derreumaux A, et al. Automatic speech analysis for the assessment of patients with predementia and Alzheimer’s disease. Alzheimer’s Dement Diagnosis, Assess Dis Monit. 2015;1: 112–124. doi: 10.1016/j.dadm.2014.11.012 27239498 PMC4876915

[25] Collins GS, Reitsma JB, Altman DG, Moons KGM. Transparent Reporting of a multivariable prediction model for Individual Prognosis Or Diagnosis (TRIPOD): The TRIPOD Statement. Ann Intern Med. 2015;162: 55. doi: 10.7326/M14-0697 25560714

[26] Konagaya Y, Washimi Y, Hattori H, Takeda A, Watanabe T, Ohta T. Validation of the Telephone Interview for Cognitive Status (TICS) in Japanese. Int J Geriatr Psychiatry. 2007;22: 695–700. doi: 10.1002/gps.1812 17431929

[27] McKhann GM, Knopman DS, Chertkow H, Hyman BT, Jack CR, Kawas CH, et al. The diagnosis of dementia due to Alzheimer’s disease: Recommendations from the National Institute on Aging-Alzheimer’s Association workgroups on diagnostic guidelines for Alzheimer’s disease. Alzheimer’s Dement. 2011;7: 263–269. doi: 10.1016/j.jalz.2011.03.005 21514250 PMC3312024

[28] American Psychiatric Association. Diagnostic and Statistical Manual of Mental Disorders, Fifth Edition. Relationship Between Physical Activity. American Psychiatric Association; 2013. doi: 10.1176/appi.books.9780890425596

[29] Kempler D, Goral M. Language and dementia: Neuropsychological aspects. Annual Review of Applied Linguistics. NIH Public Access; 2008. pp. 73–90. doi: 10.1017/S0267190508080045 21072322 PMC2976058

[30] Praat: doing Phonetics by Computer. [cited 27 Sep 2020]. Available: https://www.fon.hum.uva.nl/praat/

[31] Konig A, Satt A, Sorin A, Hoory R, Derreumaux A, David R, et al. Use of Speech Analyses within a Mobile Application for the Assessment of Cognitive Impairment in Elderly People. Curr Alzheimer Res. 2017;15. doi: 10.2174/1567205014666170829111942 28847279

[32] Chen T, Guestrin C. XGBoost: A Scalable Tree Boosting System. 2016 [cited 17 Mar 2017]. doi: 10.1145/2939672.2939785

[33] Breiman L. Random Forests. Mach Learn. 2001;45: 5–32. doi: 10.1023/A:1010933404324

[34] Hosmer DW, Lemeshow S, Sturdivant RX. Applied logistic regression. 2013. Available: https://www.amazon.co.jp/dp/B00BNFI7QK/ref=dp-kindle-redirect?_encoding=UTF8&btkr=1

[35] Kuhn M, Wing J, Weston S, Williams A, Keefer C, Engelhardt A, et al. Package “caret.” 2017 [cited 29 Oct 2017]. Available: https://cran.r-project.org/web/packages/caret/caret.pdf

[36] Chen T, Guestrin C. XGBoost: A Scalable Tree Boosting System. Proceedings of the 22nd ACM SIGKDD International Conference on Knowledge Discovery and Data Mining—KDD ‘16. New York, New York, USA: ACM Press; 2016. pp. 785–794. doi: 10.1145/2939672.2939785

[37] Adam-Bourdarios C, Cowan G, Germain-Renaud C, Guyon I, Kégl B, Rousseau D. The Higgs Machine Learning Challenge. J Phys Conf Ser. 2015;664: 072015. doi: 10.1088/1742-6596/664/7/072015

[38] Machine Learning wins the Higgs Challenge—CERN Bulletin. [cited 17 Mar 2017]. Available: http://cds.cern.ch/journal/CERNBulletin/2014/49/News Articles/1972036

[39] Desmond DW, Tatemichi TK, Hanzawa L. The Telephone Interview for Cognitive Status (TICS): Reliability and validity in a stroke sample. Int J Geriatr Psychiatry. 1994;9: 803–807. doi: 10.1002/gps.930091006

[40] DeLong ER, DeLong DM, Clarke-Pearson DL. Comparing the Areas under Two or More Correlated Receiver Operating Characteristic Curves: A Nonparametric Approach. Biometrics. 1988;44: 837. doi: 10.2307/2531595 3203132

[41] Youden WJ. Index for rating diagnostic tests. Cancer. 1950;3: 32–35. doi: 10.1002/1097-0142(1950)3:1<32::aid-cncr2820030106>3.0.co;2-3 15405679

[42] R: The R Project for Statistical Computing. [cited 22 Mar 2017]. Available: https://www.r-project.org/

[43] Filiou RP, Bier N, Slegers A, Houzé B, Belchior P, Brambati SM. Connected speech assessment in the early detection of Alzheimer’s disease and mild cognitive impairment: a scoping review. Aphasiology. Routledge; 2020. pp. 702–734. doi: 10.1080/02687038.2019.1608502

[44] Chien YW, Hong SY, Cheah WT, Yao LH, Chang YL, Fu LC. An Automatic Assessment System for Alzheimer’s Disease Based on Speech Using Feature Sequence Generator and Recurrent Neural Network. Sci Rep. 2019;9: 1–10. doi: 10.1038/s41598-018-37186-2 31862920 PMC6925285

[45] Thomas JA, Burkhardt HA, Chaudhry S, Ngo AD, Sharma S, Zhang L, et al. Assessing the Utility of Language and Voice Biomarkers to Predict Cognitive Impairment in the Framingham Heart Study Cognitive Aging Cohort Data. J Alzheimers Dis. 2020;76: 905–922. doi: 10.3233/JAD-190783 32568190

[46] Javed AR, Fahad LG, Farhan AA, Abbas S, Srivastava G, Parizi RM, et al. Automated cognitive health assessment in smart homes using machine learning. Sustain Cities Soc. 2021;65: 102572. doi: 10.1016/j.scs.2020.102572

[47] Javed AR, Sarwar MU, Beg MO, Asim M, Baker T, Tawfik H. A collaborative healthcare framework for shared healthcare plan with ambient intelligence. Human-centric Comput Inf Sci. 2020;10: 1–21. doi: 10.1186/s13673-020-00245-7

