# Supplementary material for: Dementia risks identified by vocal features via telephone conversations: A novel machine learning prediction model
Source: PLoS One. 2021 Jul 14;16(7):e0253988. doi: 10.1371/journal.pone.0253988 (PMC8279312; doi:10.1371/journal.pone.0253988)
Supplement: S1 Table — (DOCX) [file pone.0253988.s001.docx]

**S1 Table.** The questionnaire for assessing cognitive function based on TICS-J.

| Instruction | Scoring criteria | Total score |
| --- | --- | --- |
| “Please tell me your full name?” | 1pt. for first name, 1pt. for last name. | 2 |
| “What is today’s date?  “What day is today?”  “What is the current year?”  “What season are we in?” | 1pt. each for month, date, year, day of week, and season. | 5 |
| “Please tell me your address. How about postal code?” | 1pt. each for house number, street, city, prefecture, postal code. | 5 |
| “Count backwards from 20 to 1.” | 2pts. If completely correct on the first trial; 1pt. if completely correct on second trial; 0pts. For anything else. | 2 |
| “I’m going to read you a list of ten words. Please listen carefully and try to remember them. When I am done, tell me as many words as you can, in any order. Ready? The words are: cabin, pipe, elephant, coffee, face, silk, theater, watch, pillow, giant. Now tell me all the words you can remember.” | 1pt. for each correct response. No penalty for repetitions or intrusions. | 10 |
| “One hundred minus 7 equals what?”  “And 7 from that?” | Stop at 2 serial subtractions. 1pt. for each correct subtraction. | 2 |
| “What do people usually use to cut paper?”  “How many things are in a dozen?” | 1pt. for “scissor”.  1pt. for “12”. | 2 |
| “Say this: We all work together to pull the rope.”  “Say this: If the dog walks, it hits the stick.” | 1pt. for each correct repetition. | 2 |
| “Who is the prime minister of Japan right now?”  “Who is the chief cabinet secretary?” | 1pt. each for correct first and last name. | 2 |
| “With your finger, tap 5 times on the part of the phone you speak into.” | 2pts. If 5 taps are heard; 1pt. if subject taps more or less than 5 times. | 2 |
| “I’m going to give you a word and I want you to give me its opposite. For example, the opposite of hot is cold. What is the opposite of ‘west’?”  “What is the opposite of ‘convenient’?” | 1pt. for “east”.  1pt. for “inconvenient”.  *any good synonyms are accepted. | 2 |
