# Supplementary material for: Dementia risks identified by vocal features via telephone conversations: A novel machine learning prediction model
Source: PLoS One. 2021 Jul 14;16(7):e0253988. doi: 10.1371/journal.pone.0253988 (PMC8279312; doi:10.1371/journal.pone.0253988)
Supplement: S2 Table — (DOCX) [file pone.0253988.s002.docx]

**S2 Table.** Descriptive statistics of demographic and vocal data for each group on audio file basis.

| Variable | Training data,  mean (SD)  n=1,308 | Validation data,  mean (SD)  n=308 | P-value |
| --- | --- | --- | --- |
| Silent: the duration when the participant is not speaking (second) |  |  |  |
| silent_sum | 25.7 (47.1) | 25.5 (9.9) | 0.882 |
| silent_mean | 0.7 (0.4) | 0.8 (0.4) | 0.547 |
| silent_median | 0.3 (0.2) | 0.4 (0.2) | 0.006 |
| silent_minimum | 0.1 (0) | 0.1 (0) | 0.785 |
| silent_maximum | 5 (5) | 4.7 (3.7) | 0.261 |
| silent_15percentile | 0.1 (0) | 0.1 (0.1) | 0.025 |
| silent_85percentile | 1.3 (0.8) | 1.3 (0.8) | 0.180 |
| silent_standard deviation | 1.1 (1.1) | 1 (0.8) | 0.319 |
| silent_skewness | 2.4 (1.4) | 2.4 (1.2) | 0.323 |
| silent_kurtosis | 7.8 (9.7) | 7.1 (7.5) | 0.197 |
| Sounding: the duration when the participant is speaking (second) |  |  |  |
| sounding_sum | 21.1 (36.5) | 20.3 (8.2) | 0.500 |
| sounding_mean | 0.6 (0.2) | 0.6 (0.2) | 0.972 |
| sounding_median | 0.4 (0.2) | 0.5 (0.2) | 0.694 |
| sounding_minimum | 0.1 (0) | 0.1 (0) | 0.711 |
| sounding_maximum | 1.9 (0.7) | 1.9 (0.7) | 0.909 |
| sounding_15percentile | 0.2 (0.1) | 0.2 (0.1) | 0.206 |
| sounding_85percentile | 1 (0.3) | 1 (0.3) | 0.800 |
| sounding_standard deviation | 0.5 (0.2) | 0.5 (0.2) | 0.589 |
| sounding_skewness | 1.3 (0.6) | 1.3 (0.6) | 0.495 |
| sounding_kurtosis | 1.5 (2.4) | 1.7 (3) | 0.273 |
| Pitch: Voice pitch of the participant (Hz) |  |  |  |
| pitch_mean | 186.9 (36.7) | 185.9 (34.1) | 0.640 |
| pitch_median | 173.5 (42.3) | 170.3 (39.8) | 0.209 |
| pitch_minimum | 70.3 (31.3) | 69 (30.3) | 0.499 |
| pitch_maximum | 538.6 (72) | 543.4 (69) | 0.277 |
| pitch_15percentile | 142.6 (35) | 141.1 (32.4) | 0.468 |
| pitch_85percentile | 228.3 (57.2) | 228.4 (52.5) | 0.968 |
| pitch_standard deviation | 63.1 (23.1) | 65.2 (22) | 0.137 |
| pitch_skewness | 2.6 (1.5) | 2.6 (1.5) | 0.952 |
| pitch_kurtosis | 13.4 (14) | 12.5 (13) | 0.249 |
| Pitch difference: Amount of change in voice pitch every 0.01 seconds (Hz) |  |  |  |
| pitch_d_mean | -0.4 (1) | -0.3 (1) | 0.004 |
| pitch_d_median | -1 (0.5) | -0.9 (0.5) | 0.049 |
| pitch_d_minimum | -156.1 (96.9) | -159.8 (101.4) | 0.565 |
| pitch_d_maximum | 225.6 (112.2) | 228.8 (112.2) | 0.657 |
| pitch_d_15percentile | -5.3 (1.6) | -4.8 (1.5) | <0.001 |
| pitch_d_85percentile | 3.2 (1.5) | 3 (1.4) | 0.016 |
| pitch_d_standard error | 16.9 (7.1) | 17.3 (7.6) | 0.407 |
| pitch_d_skewness | 4.1 (6.4) | 3.9 (6.4) | 0.725 |
| pitch_d_kurtosis | 128.9 (93.5) | 126.1 (86.1) | 0.603 |
| Intensity: Voice intensity of the participant (dB) |  |  |  |
| intensity_mean | 75.2 (4.6) | 75.3 (6.2) | 0.676 |
| intensity_median | 76.9 (5.2) | 77 (6.9) | 0.761 |
| intensity_minimm | 34.3 (14.9) | 36.6 (7.4) | <0.001 |
| intensity_maximum | 89.3 (3.5) | 89.3 (4.3) | 0.940 |
| intensity_15percentile | 67.1 (4.1) | 67.2 (5.6) | 0.718 |
| intensity_85percentile | 83.2 (4.9) | 83.4 (6.3) | 0.578 |
| intensity_standard deviation | 8.6 (1) | 8.6 (1) | 0.932 |
| intensity_skewness | -1.3 (0.6) | -1.2 (0.4) | 0.001 |
| intensity_kurtosis | 2.9 (13.8) | 2.1 (1.4) | 0.029 |
| Intensity difference: Amount of change in voice intensity every 0.01 seconds (dB) |  |  |  |
| intensity_d_mean | 0 (0) | 0 (0) | 0.906 |
| intensity_d_median | -0.1 (0) | -0.1 (0) | <0.001 |
| intensity_d_minimum | -11.2 (12.3) | -10.7 (2.2) | 0.255 |
| intensity_d_maximum | 15.4 (11.7) | 14.4 (2.5) | 0.004 |
| intensity_d_15percentile | -1.8 (0.3) | -1.8 (0.4) | 0.010 |
| intensity_d_85percentile | 1.6 (0.3) | 1.6 (0.4) | 0.738 |
| intensity_d_standard deviation | 2.6 (0.4) | 2.6 (0.4) | 0.038 |
| intensity_d_skewness | 0.7 (0.4) | 0.7 (0.3) | 0.002 |
| intensity_d_kurtosis | 6 (38.9) | 4.6 (1.9) | 0.187 |
| Spectrum (Hz) |  |  |  |
| spectrum_center of gravity | 581.5 (140.8) | 644 (178) | <0.001 |
| spectrum_standard deviation | 440.8 (134.2) | 491.2 (149.1) | <0.001 |
| spectrum_skewness | 3.3 (1) | 2.8 (1) | <0.001 |
| spectrum_kurtosis | 16 (11.6) | 11.7 (8.9) | <0.001 |
